# Pulmonary rehabilitation improves survival in patients with idiopathic pulmonary fibrosis undergoing lung transplantation

**DOI:** 10.1038/s41598-019-45828-2

**Published:** 2019-06-27

**Authors:** Juliessa Florian, Guilherme Watte, Paulo José Zimermann Teixeira, Stephan Altmayer, Sadi Marcelo Schio, Letícia Beatriz Sanchez, Douglas Zaione Nascimento, Spencer Marcantonio Camargo, Fabiola Adélia Perin, José de Jesus Camargo, José Carlos Felicetti, José da Silva Moreira

**Affiliations:** 1 Postgraduate Program in Pulmonology, Universidade Federal do Rio Grande do Sul, Porto Alegre, Brazil; 2 Department of Lung Transplantation, Santa Casa de Misericordia de Porto Alegre, Porto Alegre, Brazil; 3 Pulmonary Rehabilitation Program, Santa Casa de Misericordia de Porto Alegre, Porto Alegre, Brazil; 4 Departament of Medicine, Universidade Federal de Ciências da Saúde de Porto Alegre, Porto Alegre, Brazil; 5 Medical Imaging Research Laboratory, Santa Casa de Misericordia de Porto Alegre, Porto Alegre, Brazil

**Keywords:** Respiratory tract diseases, Prognosis

## Abstract

This study was conducted to evaluate whether a pulmonary rehabilitation program (PRP) is independently associated with survival in patients with idiopathic pulmonary fibrosis (IPF) undergoing lung transplant (LTx). This quasi-experimental study included 89 patients who underwent LTx due to IPF. Thirty-two completed all 36 sessions in a PRP while on the waiting list for LTx (PRP group), and 53 completed fewer than 36 sessions (controls). Survival after LTx was the main outcome; invasive mechanical ventilation (IMV), length of stay (LOS) in intensive care unit (ICU) and in hospital were secondary outcomes. Kaplan-Meier curves and Cox regression models were used in survival analyses. Cox regression models showed that the PRP group had a reduced 54.0% (hazard ratio = 0.464, 95% confidence interval 0.222–0.970, p = 0.041) risk of death. A lower number of patients in the PRP group required IMV for more than 24 hours after LTx (9.0% vs. 41.6% p = 0.001). This group also spent a mean of 5 days less in the ICU (p = 0.004) and 5 days less in hospital (p = 0.046). In conclusion, PRP PRP completion halved the risk of cumulative mortality in patients with IPF undergoing unilateral LTx

## Introduction

Idiopathic pulmonary fibrosis (IPF) is a non-reversible fibrotic lung disease characterized by a progressive decline of lung function, with a largely unpredictable clinical course and a median survival time of 2–3 years from diagnosis^1,2^. The progressive morphological changes that IPF causes in the lung can lead to respiratory failure and death, especially after exacerbation episodes. Lung transplantation (LTx) is a well-documented intervention that prolongs survival in patients with IPF^3^.

A recent meta-analysis showed the benefits of pulmonary rehabilitation with exercise training for interstitial lung diseases (ILD) among patients both on and off the waiting list for LTx^4^. Also, Dowman *et al*. also demonstrated clinically important improvements in 6-minute walking distance (6MWD), symptoms, and HRQoL following exercise training^4,5^. The role of pulmonary rehabilitation program (PRP) with physical exercise prior to lung transplant in patients with ILD is already established in previous studies^6–9^. PRP is an important tool to boost physical function prior to transplant and to accelerate the recovery after the procedure^4^.

Despite the proven benefits of exercise training for patients with ILD, the role of PRP in survival after LTx for patients with IPF remains unknown. Thus, our primary goal was to evaluate whether a pulmonary rehabilitation program (PRP) is independently associated with improved survival in patients with IPF undergoing LTx. Also, we analyzed the impact of PRP to reduce in-hospital morbidity after LTx.

## Methods

### Study sample

This retrospective quasi-experimental study included patients with IPF who underwent unilateral LTx between January 2007 and June 2015. IPF was diagnosed by multidisciplinary group discussions based on high-resolution computed tomography and/or surgical lung biopsy before LTx, or in the explanted lung showing a UIP pattern according to the 2011 ATS/ERS/JRS/ALAT guidelines^2^.

**Figure 1:**
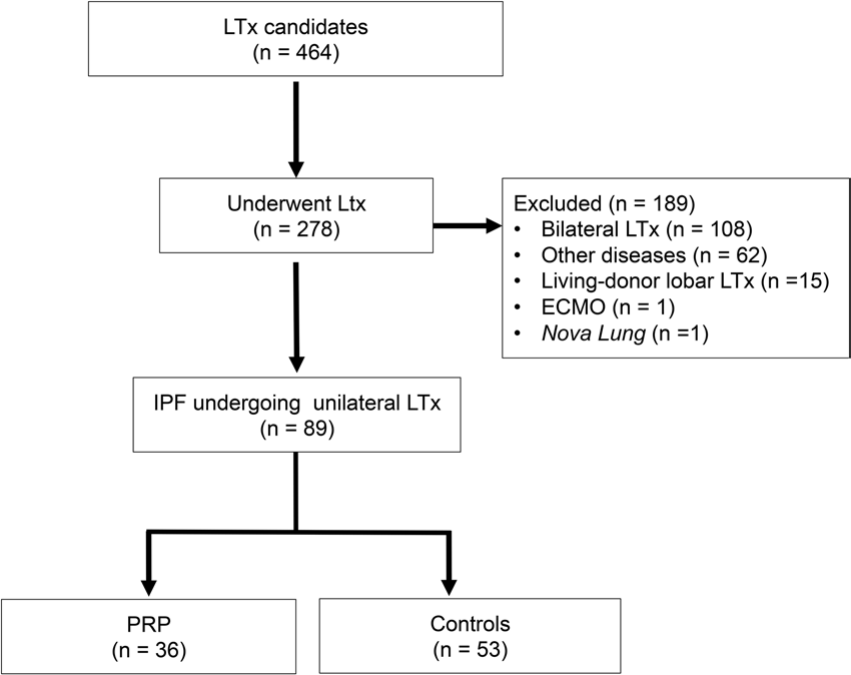
Flowchart of the study. LTx: lung transplantation; ECMO: extracorporeal membrane oxygenation; IPF: idiopathic pulmonary fibrosis; PRP: pulmonary rehabilitation program.

Of the 464 consecutive LTx candidates who were referred to our quaternary medical center and subsequently listed for LTx, 278 underwent the procedure during the defined period. We then excluded patients with diseases other than IPF, those with bilateral LTx, those who received living donor lobar LTx, those who required invasive mechanical ventilation (IMV) (*Nova Lung*®) and/or extracorporeal membrane oxygenation (ECMO) before LTx. (Fig. 1), leaving an analytical sample of 89 patients with IPF who were selected for unilateral LTx according to international guidelines^10^. Thirty-six of these patients completed at least 36 sessions in a PRP while on the waiting list for LTx (PRP group), and 53 completed fewer than 36 sessions and were considered controls. This study was approved by the Hospital Santa Casa de Misericordia de Porto Alegre human ethics committee/internal review board with waiver of consent.

### Pulmonary rehabilitation program before lung transplantation

The PRP consisted of medical appointments with the transplant team every 2 months, psychiatric evaluations, nutritional counseling, social assistance, and monthly educational lectures. The physical training component of the PRP was administered by two physical therapists, with sessions three times a week, totaling 36 sessions. During this physical training, patients performed a warm-up, muscle strengthening, and aerobic exercises. The warm-up consisted of breathing exercises (respiratory cycle) associated with arm raising. Muscle strengthening was based on arm and leg exercises with an initial load of 30% of one repetition maximum testing and then one set of 10 repetitions per exercise. The load was increased by 0.5 kg every seven sessions according to the patients’ tolerance. Aerobic exercises were performed on a treadmill, beginning at 70% of the speed of the patient on the 6MWD test, with a progressive protocol every 6 minutes for the variable time until 30 minutes was achieved. The speed was increased by 0.3 km/h every seven sessions. The completion of all exercises was limited when the patient reported dyspnea or leg fatigue, indicated by a modified Borg scale score greater than 4, and when the SpO_2_reached 92%. When patients presented a SpO2 < 92%, the exercise was not stopped, but the intensity was reduced, and the oxygen flow was increased as an attempt to maintain the effort and incentive the patient to tolerate dyspnea. At the end of each session, patients performed stretching for all the major muscle groups they had worked. During the PRP, all patients received continuous oxygen therapy in accordance with their medical prescriptions, and they were constantly monitored by pulse oximetry to maintain a SpO_2_ ≥ 92%. The modified Borg scale was used for measuring dyspnea and leg discomfort.

### Treatment received after lung transplantation

After LTx, patients were prescribed maintenance immunosuppressive therapy based on the concomitant use of three drugs: comprising cyclosporine or tacrolimus, azathioprine or mycophenolate, and corticosteroids. In some situations, like drug toxicity, patients were switched to an alternative immunosuppressive regimen^11^. The patients performed modified preemptive prophylaxis for cytomegalic infection^12^ and antifungal prophylaxis with inhaled amphotericin or voriconazole according to risk stratification^13^.

### Pulmonary function tests and questionnaires

Pulmonary function tests were performed at baseline (when the patient was included on the wait list for LTx, before the 36 sessions of PRP) and after the 36 sessions of PRP in accordance with the technical procedures and the acceptability and reproducibility criteria of the American Thoracic Society/European Respiratory Society and the Brazilian Thoracic Association (BTA)^14–16^. All pulmonary function tests were performed in the pulmonary function laboratory of our institution, which is a laboratory certified by the BTA. In addition to pulmonary function tests, the same physical therapists administered the 6MWD test, in accordance with the recommendations of the American Thoracic Society^17^, and the *Medical Outcomes Study 36-item Short Form Health Survey* (SF-36)^18^ to evaluate HRQoL.

### Data analysis

The primary outcome was survival after LTx. Secondary outcomes were days on IMV, LOS in ICU and LOS in hospital after LTx. Data are presented as absolute and percentage frequencies, mean ± standard deviation (95% confidence interval) or median (interquartile range). The normal distribution of the database was evaluated through the Shapiro-Wilk test. Comparisons of proportions were evaluated by the Chi-square test for categorical variables and the Student’s t-test for continuous variables. Kaplan-Meier curves, compared to log-rank tests, were used for cumulative survival analyzes. P-values < 0.05 were considered significant. Survival analysis was performed using Cox proportional risk regression models: (i) events were defined as time to death; (ii) censored data were used when the event did not occur at the end of the follow-up period. All parameters with a p-value < 0.10 in the univariate analysis were included in a multivariate model and considered statistically significant if the overall p-value was <0.05. The analysis supported the hypothesis of proportional risk. All analyses were performed in the Statistical Package for the Social Sciences software (PASW Statistics for Windows, Version 18.0, SPSS Inc., Chicago, IL, USA).

## Results

There were no significant differences observed at baseline between the characteristics of the PRP group and controls, with the exception of the 6MWD (327.92 ± 140.78 vs. 404.78 ± 107.79, p = 0.007) (Table 1). After completion of 36 sessions of PRP, the PRP group showed a significant increase in 6MWD (∆ 43 ± 86 m, p = 0.005) and an improvement in four domains of SF-36 (physical functioning: ∆ 10 ± 26, p = 0.025; role physical: ∆ 24 ± 31, p = 0.000; vitality ∆: 13 ± 24, p = 0.002; role emotional: ∆ 26 ± 47, p = 0.002). The control group completed an average of 10 (range 7–25) sessions of PRP.

**Table 1:** Characteristics of the study sample at baseline, i.e., upon placement on the wait list for lung transplantation.

Variables	Total (N = 89)	Control (n = 53)	PRP (n = 36)	*p*
Male	57 (64%)	32 (60.3%)	25 (69.4%)	0.500
Age, y	55.93 ± 10.93	56.79 ± 10.84	54.67 ± 11.08	0.958
BMI, kg/m^2^	25.42 ± 3.870	25.44 ± 4.110	25.39 ± 3.550	0.371
FEV_1_, L	1.33 ± 0.54	1.26 ± 0.51	1.44 ± 0.56	0.119
FVC, L	1.61 ± 0.50	1.56 ± 0.47	1.69 ± 0.54	0.220
FEV_1_, %	46.16 ± 15.23	43.76 ± 15.02	49.65 ± 15.07	0.078
FVC, %	44.33 ± 12.55	43.70 ± 12.14	45.25 ± 13.25	0.570
FEV_1_/FVC	82.33 ± 21.43	81.22 ± 24.05	83.97 ± 17.03	0.556
PASP, mmHg	45.80 ± 15.55	46.37 ± 16.50	44.97 ± 14.25	0.682
6MWD, meters	359.36 ± 133.18	327.92 ± 140.78	404.78 ± 107.79	0.007
Oxygen flow, L/min	5.19 ± 1.65	5.02 ± 1.40	5.52 ± 2.04	0.206
Median time in list, months	5.1 [2.4–10.7]	2.7 [1.4–10.7]	7.6 [4.5–11.4]	0.133
Follow-up time, years	2.1 [0.3–4.1]	1.9 [0.1–3.6]	2.7 [0.5–4.5]	0.405

Note: Data presented as number (frequency), mean ± standard deviation or median [interquartile range].

PRP: pulmonary rehabilitation program, BMI: body mass index, FEV1: forced expiratory volume in the first second, FVC: forced vital capacity, PASP: pulmonary artery systolic pressure, 6MWD: 6-minute walking distance test, IPF: idiopathic pulmonary fibrosis.

**Figure 2:**
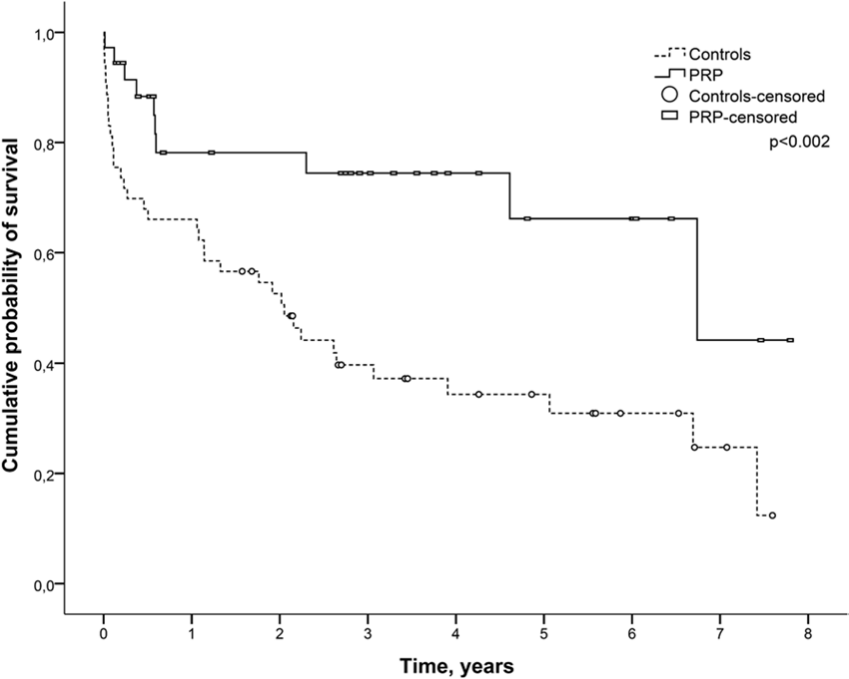
Kaplan-Meier curves of cumulative survival of patients stratified by PRP status after lung transplantation. PRP = pulmonary rehabilitation program.

Cumulative survival in the PRP group during the follow-up period (89.9%) was greater than that of controls (62.9%), as observed in the Kaplan-Meier curve (p = 0.002) (Fig. 2). IMV for more than 24 hours was associated with higher mortality in the PRP group, whereas PRP was a protective factor in the univariate analysis (Table 2). After adjusting for IMV for more that 24 hours, the Cox regression model showed that the PRP group had a 54.0% (HR = 0.464, 95% CI: 0.222–0.970, p = 0.041) reduced risk of death when compared to controls. Moreover, IMV for more than 24 hours after LTx presented an 88.0% greater risk of death when compared to those who remained on IMV for less than 24 hours, after adjustment (HR = 1.881, 95% CI: 1.009–5.308, p = 0.047).

**Table 2:** Cox regression analysis for mortality after unilateral lung transplantation (N = 89).

Variables	Crude analysis HR (95% CI)^a^	*p*	Adjusted analysis HR (95% CI)^b^	*p*
PRP	0.352 (0.176–0.703)	0.003	0.464 (0.222–0.970)	0.041
Male	1.151 (0.626–2.115)	0.517		
Age, y	1.007 (0.977–1.037)	0.630		
BMI, kg/m^2^	0.972 (0.977–1.003)	0.526		
FEV_1_, %	0.979 (0.959–1.000)	0.130		
FVC, %	0.987 (0.959–1.015)	0.373		
FEV_1_/FVC	0.991 (0.987–1.003)	0.175		
6MWD, meters	0.998 (0.996–1.001)	0.314		
Oxygen flow, L/min	1.083 (0.913–1.286)	0.357		
Time on waiting list, days	1.000 (0.977–1.001)	0.741		
IMV > 24 hours	2.551 (1.400–4.650)	0.002	1.881 (1.009–5.308)	0.047
LOS in ICU, days	1.002 (0.975–1.030)	0.851		
LOS in hospital, days	1.004 (0.987–1.020)	0.627		

Note: ^a^All parameters at a significance level of p-value less than 0.10 in the univariate analysis were included in a multivariable model; ^b^Model adjusted for PRP and IMV time > 24 hours.

PRP: pulmonary rehabilitation program, HR: hazard ratio, CI: confidence interval, BMI: body mass index, FEV1: forced expiratory volume in the first second, FVC: forced vital capacity, PSAP: pulmonary artery systolic pressure, 6MWD: 6-minute walk distance, IMV: invasive mechanical ventilation, ICU: intensive care unit, LOS: length of stay.

Regarding the secondary outcomes, fewer patients in the PRP group remained in IMV for more than 24 hours after LTx compared to controls (9.0% vs. 41.6% p = 0.001) (Table 3). The PRP group also had a shorter LOS in the ICU (5 days vs. 7 days, p = 0.004) and a shorter LOS in hospital compared to controls (20 days vs. 25 days, p = 0.046). Moreover, the PRP group had a lower mortality during their ICU stay (p = 0.006), and a higher survival rate 5 years after LTx (89.9% vs. 60.9%, p < 0.001).

**Table 3:** Primary and secondary outcomes after unilateral lung transplantation.

Variables	Total (N = 89)	Control (n = 53)	PRP (n = 36)	*p*
IMV > 24 hs.	45 (50.6%)	37 (69.8%)	8 (22.2%)	0.001
Days in ICU	6 [4.5–13]	7 [5–19]	5 [4–7.5]	0.004
Days in hospital	23 [19–33]	25 [20–39]	20 [17.7–26]	0.046
**Mortality**				
ICU	18 (20.2)	16 (18.0)	2 (2.2)	0.006
1 year after LTx	25 (28.1)	18 (20.2)	7 (7.9)	0.156
5 years after LTx	42 (47.2)	33 (37.1)	9 (10.1)	<0.001
Total study time	46 (51.7)	36 (40.4)	10 (11.2)	<0.001

Note: Data presented as number (frequency), mean ± standard deviation or median [interquartile range].

PRP, pulmonary rehabilitation program; ICU: intensive care unit, LTx: lung transplantation.

## Discussion

This study aimed to investigate the contribution of a PRP to the reduction of mortality after unilateral LTx in patients with IPF. We found that completion of a PRP halved the risk of death after LTx in patients with IPF even after adjusting for prolonged IMV. Patients in the PRP group also benefited from less time on IMV and a reduced LOS in the ICU and in hospital.

Patients with extended LOS following lung transplantation more often receive IMV or ECMO for a prolonged time and are also at a higher risk to be colonized by multidrug-resistant bacteria^19^. In our study, only 9.0% of those in the PRP group remained in IMV for more than 24 hours (vs. 41.6% in the control group), which demonstrates the importance of the PRP for the recovery of those patients with IPF undergoing unilateral LTx, in accordance to previous studies in the literature^20,21^.

Within our knowledge, this is one of the few studies to evaluate the impact of a PRP program in the mortality of patients with IPF after LTx. Our group already demonstrated that multidisciplinary PRP was helpful for patients on the waiting list for LTx, leading to improvements in 6MWD and quality of life^22^. However, previously identified risk factors for early mortality such as age (<55 years), sex (male sex above 65 years) and single-lung transplants^22–24^ were not associated with prediction of mortality after LTx in our sample. This finding may be related to the fact that the majority of individuals in our sample were under 65 years. Also, there was no significant difference for patients with or without oxygen rate prescription or increased rate during workout regimen.

The control group completed an average of 10 sessions of the program. Main reasons for absence were due to logistic problems (patients referred to our center come from different regions of the country, and are more likely to miss a session due to delays in transportation) or if the patient underwent LTx before completion of the 36 sessions of PRP. Even though patients were required to have completed at least 75% of sessions to be included in the PRP group, all patients from this group completed all 36 sessions scheduled.

All included patients were submitted to unilateral LTx. This is a common practice in our country and institution^25^. The major reason behind this is due to the scarcity of organs donor in the nation; therefore, the supply is not enough to meet the demand of individuals on waiting list. For international centers, bilateral transplantation is preferred as it is associated with better outcomes compared to patients undergoing unilateral LTx^26^. We had only a few cases of bilateral procedures for IPF, but those cases were not included in the sample as they would probably have better outcomes and result in an additional source of bias. This is an important limitation of our paper; future investigations should aim to include patients undergoing bilateral LTx as well.

Our study has other limitations, including its quasi-experimental design. Randomization in terms of completion of a PRP is quite difficult now that it has become a well-recognized form of treatment for chronic respiratory diseases, and improvement in physical conditioning has shown to be beneficial for patients pre-transplant. Thus, the design and lack of randomization could be potential sources of selection bias. However, the two groups were homogeneous in regard to most baseline characteristics, except for baseline 6WMD – which was not related to survival benefit in the main analysis. Other variables not included in the present study (e.g., donor variables, cardiopulmonary exercise test, etc.) were already shown to predict survival in the literature and were not available for the present study.

In conclusion, completion of a PRP halved the risk of cumulative mortality in patients with IPF undergoing unilateral LTx, after adjusting for prolonged time (>24 hours) on IMV. Moreover, the PRP group had a reduced risk of prolonged IMV, LOS in the ICU, and total LOS in hospital. Further studies including other recipient diseases and donor variables should be performed in order to confirm PRP as an independent predictor of survival in all kinds of recipients.

## Data Availability

The dataset is fully available from the corresponding author on reasonable request.
